# Birefringent Glass‐Engraved Tilted Pillar Metasurfaces for High Power Laser Applications

**DOI:** 10.1002/advs.202301111

**Published:** 2023-06-19

**Authors:** Nathan J. Ray, Jae‐Hyuck Yoo, Hoang T. Nguyen, Michael A. Johnson, Eyal Feigenbaum

**Affiliations:** ^1^ Lawrence Livermore National Laboratory Livermore CA 94550 USA

**Keywords:** anisotropic surface layer, angled etching, birefringence, metasurface, thin film dewetting

## Abstract

Birefringent materials—which are highly needed in high power laser systems—may be limited in usage due to the laser‐induced damage threshold of traditional birefringent materials. This work reports here on all‐glass metasurfaces, fabricated by angled etching through sacrificial metal nanoparticle (NP) etching masks, for generation of effective birefringence in the formed layer. As a result, a fused silica metasurface, monolithic to the underlying substrate, is demonstrated to exhibit a birefringence of 6.57° under 375 nm illumination. Full‐wave analysis shows a good agreement with the measurement and presents potential paths forward to increasing the effective metasurface birefringence. This is the first demonstration, to the best of knowledge, of an etching technique to obtain the resulting tilted pillar‐like nanofeatures. The anisotropy of the metasurface nanoelements along the two window in‐plane major axes presents different effective paths for the two polarizations and thus generates birefringence in a nonbirefringent material. Additionally, the imparted anisotropy lends itself to manipulation of physical properties of the surface as well, with metasurface feature orientation suppressing water flow along one principal axis and giving rise to water flow steering capabilities.

## Graphical abstract

A fabrication process is described for generation of angled metasurface features. Resultant anisotropic index of refraction enables nonbirefringent materials to exhibit birefringence, demonstrated here with fused silica under illumination by a 375 nm source. A simulation‐informed roadmap is provided for quarter‐wave plates using this technology. Additionally, the imparted anisotropy lends itself to manipulation of physical properties of the structure as well, with orientation suppressing water flow along one principal axis and giving rise to the capability of water flow steering.

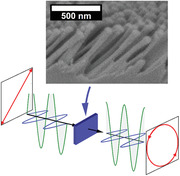

## 1 Introduction

Birefringence, a phenomenon traditionally associated with noncubic crystalline structures^[^ ^1^, ^2^, ^3^ ^]^ in which light propagates at a different velocity depending on the vibration plane, is the result of optical anisotropy of the index of refraction. Thus, the light propagation may differ between its two polarization components in the ordinary (o) and the extraordinary (e) directions, corresponding to the ordinary refractive index *n*_o_ and extraordinary refractive index, *n*_e_. The difference of these two, |*n*_o_ – *n*_e_|, defines the birefringence of the material. It is not surprising, then, that birefringence plays an important role in optical systems, with applications including, but not limited to, polarization microscopy,^[^ ^4^, ^5^ ^]^ optical isolators,^[^ ^6^, ^7^, ^8^ ^]^ and polarization cleanup following reflections from metal or dielectric mirrors.^[^ ^9^, ^10^ ^]^

One potential limitation of these optical elements is that they inevitably introduce a maximum permissible fluence on the system they are in—these components will damage under exposure to high laser fluences. Consequently, their application space has been limited to lower energies. However, waveplates are useful with high power lasers, as attenuation elements that are not absorption‐based (such as polarization beam splitters) and waveplate rotators, if available, could open new possibilities for these systems. A survey of commercially available quarter‐wave plates reveals that, while dependent upon the materials used (i.e., polymer versus crystalline), the laser‐induced damage threshold (LIDT) is generally lower than ≈8 J cm^−2^ for a 5 ns pulse at 355 nm. These reported LIDT values will be reduced further if a larger beam is used, as larger beams are more likely to illuminate defects and reveal the “true” damage threshold. This presents a core challenge for high energy laser systems such as the National Ignition Facility (NIF),^[^ ^11^ ^]^ where a common practice is to use fused silica optics (apertures up to meter length scales) that initiate damage above 20 J cm^−1^.^[^ ^2^, ^12^ ^]^

Recently, birefringence has been demonstrated in subwavelength gratings^[^ ^13^, ^14^, ^15^, ^16^, ^17^ ^]^ and in layers generated by glancing‐angle deposition (GLAD) of silica and other metal oxides.^[^ ^18^, ^19^, ^20^ ^]^ We focus here on the latter, as it is technologically challenging to generate short‐period gratings over large apertures. The advantage of the GLAD process, which fabricated a quarter‐wave plate consisting of 31 layers of SiO_2_ with alternating deposition angle ±*θ*, is that a large bandgap material (silica) was used to yield a large LIDT of 12.5 J cm^−2^ (1‐on‐1 testing; 0.4 mm diameter; 1 ns pulse; 351 nm).^[^ ^18^ ^]^ While this result is an ≈50% increase in LIDT over the best performing commercially available waveplates available today, it stands to reason that some of the damage was generated by the “build‐up” process that was used to fabricate 31 layers. For instance, a single sol‐gel layer (colloidal silica) on fused silica has been shown to damage at ≈37 J cm^−2^ (355 nm, 7.5 ns, 0.58 mm 1/e^2^ diameter, 10 Hz),^[^ ^21^ ^]^ which is more than twice that of the alternating GLAD layers when scaling for the changes in pulse duration and beam diameter. Relative to sol‐gel layers, potential sources for the decrease of the LIDT with GLAD birefringence is the usage of 31 silica layers, i.e., deposited silica versus bulk fused silica glass, and 31 material interfaces. Furthermore, nanobubbles and voids have been shown to contribute to the LIDT for multilayer dielectric coatings,^[^ ^22^ ^]^ and a similar mechanism could be factoring in during GLAD processing as the substrate is rotated between the two deposition angles, in turn causing the resultant layers to exhibit a lower LIDT than fused silica.

An alternative approach to this additive fabrication technique, and one we will demonstrate here, is forming a metasurface (MS) by engraving fused silica, i.e., removing material by etching into the substrate. In doing so, the fabricated structure is monolithic to the underlying robust fused silica and there are no added materials or additional interfaces. The end‐result of this processing is an anisotropic surface layer, incorporating optical and physical anisotropy (i.e., birefringence and surface energy properties) in a material that does not exhibit bulk anisotropy (i.e., no native birefringence). While there are lithographic techniques to accomplish glass‐engraving by carefully etching each metasurface feature individually,^[^ ^23^, ^24^, ^25^ ^]^ we will focus here on fabrication processes that are compatible with large aperture applications. Two substrate‐engraving techniques based on directional dry etch that have demonstrated potential for scaling up to meter‐length scale optics apertures while maintaining few tens of nanometers period scale are: (1) ion bombardment or the creation of in situ nucleation sites,^[^ ^26^, ^27^, ^28^ ^]^ and (2) etching masks to guide the etching process.^[^ ^29^, ^30^, ^31^, ^32^ ^]^ The result of dry etching in normal incidence are metasurfaces with randomly distributed nanofeatures, yet with a well‐controlled and predictive distribution of properties that determine the effective optical properties of the layer from mixing formula rules of the constituents (i.e., glass features and air vacancies). In this work we modify technique (2), since being a mask‐based method, it enables the formation and control over the nanoparticle (NP) mask based on prior knowledge, and anisotropy is obtained by tilting the incidence angle of the ion beam with respect to the mask. Moreover, this technique with normal incidence etching through NP masks has previously demonstrated a laser‐induced damage onset for 351 nm laser exposure at roughly 30 J cm^−2^.^[^ ^29^ ^]^

Here, we present a method to form tilted nanopillar‐based substrate‐engraved metasurfaces leading to tailorable effective birefringence applications. These MS layers were generated by angled etching through a sacrificial self‐generated NP ensemble functioning as an etching mask. We demonstrate a polarization retardation rotation angle of 6.57° using this method, validated with full‐wave simulations predicting 6.69°, and further utilize the model to present potential pathways to further increase the effective birefringence. We will also show that the tilted nanopillars modify the physical surface energy properties to present strong anisotropy, such that water preferentially flows along one principal axis.

## 2 Optical Anisotropy: Birefringence

**Figure 1:**
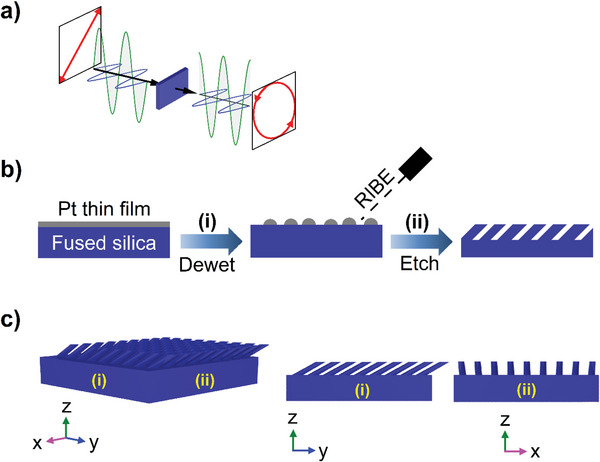
Birefringent metasurface fabrication process and resultant structure. Polarization rotation when linearly polarized light propagates through a birefringent medium and is converted to circular polarization. a) Thin Pt film dewet on fused silica substrate (step i) and subsequently etched at an angle by reactive ion beam etching (RIBE) (step ii), b) 3D depiction (left), and at the two orthogonal principal planes identified as (i) and (ii), center and right (c).

The concept of polarization retardation rotation is depicted in **Figure** **1**a for an example case where a quarter‐wave plate is converting the light from a linear polarization to a circular polarization. To accomplish this rotation by using a MS on a nonbirefringent material, the symmetry of the structure on the surface plane must be broken. In this work, fabrication of substrate‐engraved birefringent MS was done by angled etching through metal NP etching masks generated atop the substrate, see Figure 1b. Fused silica was used as the underlying substrate material due to its abundant use in optical systems and relatively high laser‐damage threshold. The etching masks were generated through solid‐state diffusional dewetting (SSD dewetting) of Pt thin films, see Figure 1b step (i) for a depiction, where SSD dewetting is a process by which thin films will self‐assemble into ensembles of particles during annealing. In dewetting, which has been investigated previously for gold on fused silica,^[^ ^33^ ^]^ the ensemble particle center‐to‐center mean spacing (period) and mean particle size increase with increasing initial metal film thickness; conversely, for a sufficiently thin initial film the resultant particle ensemble has size and period on the nanoscale. In this work, platinum is used rather than gold due to the increased etching selectivity of Pt relative to Au, i.e., Pt enables deeper etching.

To fully exert control over the etching mask, seeded dewetting may be necessary. In conventional SSD dewetting the annealing temperature provides another lever of control, as this temperature permits tunability of the NP size and period. However, the NP size and period remain largely coupled together and cannot be controlled independently. Seeded dewetting can be used to orthogonalize the control over NP size and period, where the initial metal film thickness and dewetting temperature are selected to generate the desired NP size and period of the “seed” NP distribution, and subsequent iterations of thin film deposition/dewetting atop the initial NP ensemble “seed” induces material accumulation at those seeded locations—modifying the size but not the period.^[^ ^34^ ^]^ These techniques have been demonstrated previously for a fused silica/gold system, and the fabrication process used here to generate platinum etching masks is given in greater detail in Supporting Information.

Following assembly of the Pt etching mask, reactive ion beam etching (RIBE) was used to transfer the mask pattern to the substrate, where RIBE is a process by which etching is obtained by directing a beam of reactive ions toward the substrate, see Figure 1b, step (ii). Benefits of using RIBE include the ease of which angled etching can be completed by rotation of the beam and/or sample, in addition to removing the complications associated with etching thick glass substrates that may plague reactive ion etching, RIE (which requires an electric field to run through the substrate being etched, here insulating glass). For this work, etching was done with the ion beam making an angle of 48° with respect to the substrate normal. Details pertaining to the etch recipe used are given in Supporting Information. Termination of the etch prior to mask material removal results in slanted cylinders, etching to mask depletion results in slanted cones, and etch termination between those two conditions leads to controllable truncated cones. A cross‐section depiction of a MS composed of slanted cylinders is depicted in Figure 1b following step (ii). After this etching step, residual Pt masking material was removed by soaking the optic in an aqua regia bath; consequently, the fabricated MS consists of a glass‐only structure. If a different substrate material is chosen to be compatible in the infrared (IR) and the MS feature sizes are scaled appropriately, these structures may introduce enhanced IR absorption as was documented elsewhere.^[^ ^35^ ^]^

**Figure 2:**
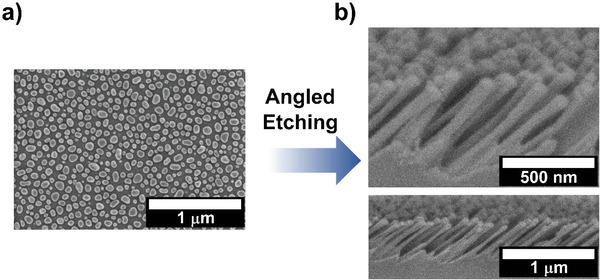
Etching mask and resultant etched metasurface with angled features. Etching mask (top view) (a), and resultant structure after etching (plane i (*y*–*z*) in Figure 1) (b).

Extending the etched cross‐section view depicted in Figure 1b to three‐dimensional space, see Figure 1c, gives a more insightful visualization of these birefringent surfaces. The structure portrayed in Figure 1c is an idealized case, i.e., an array of identical and periodically repeating MS features. An off‐axis viewing angle is shown in Figure 1c on the left, where two orthogonal planes are identified: plane (i) is in the family of planes that includes the feature tilt, referring to Figure 1b for a simplistic view, and plane (ii) represents the family of planes perpendicular to plane (i) in which the features tilt out of the page. Looking more closely at these orthogonal cross‐sections, see Figure 1 planes (i) and (ii), it is apparent that light polarized along the two principal axes of the structure (*x*–*z* and *y*–*z* directions) observes a different metasurface structure and optical effective index, producing effective birefringence. This technology can be married to past antireflective work with vertical etching of similar masks, where it was observed that, similar to uniform films, the observed reflectance is harmonically dependent on the MS layer depth.^[^ ^36^, ^37^ ^]^ Reflectance is expected to be polarization dependent due to index birefringence, and can be found as a function of incident wavelength in Figure S3 (Supporting Information). Through the described process of dewetting and RIBE, a MS was fabricated with a layer thickness *L* = 450 ± 20 nm and a mask NP center‐to‐center spacing (period) of 101 ± 25 nm; the etching mask and resultant structure are displayed as electron micrographs in **Figure** **2**. The etched structure, see Figure 2b, presents viewing plane (i) of the anisotropic structure depicted in Figure 1c. Moreover, every step in this fabrication process is scalable, which is essential for applications requiring large aperture optics. The extinction spectrum of this structure is given in Figure S4 (Supporting Information). Reduction of the etching mask NP spacing, as demonstrated in Figure S1 (Supporting Information), is expected to reduce scattering at the shorter wavelengths.

**Figure 3:**
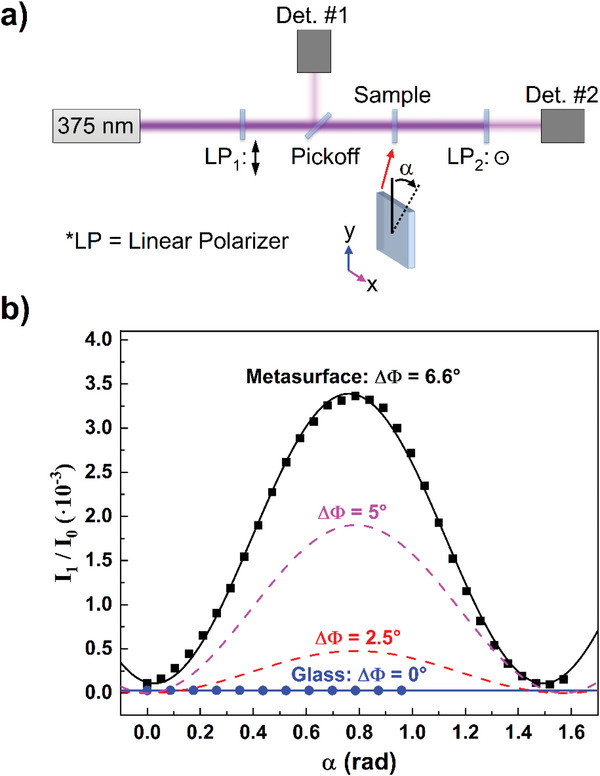
Optical setup for birefringence measurement and measured data. Measurement setup (a), and measured data (b). The measured data for the generated metasurface (MS) and a reference piece of fused silica glass are depicted by the black and blue data, respectively. Equation (1) is used to fit the two datasets and is also shown for phase retardation (Δ*Φ*) values of 2.5° and 5° to illustrate the trend of increasing phase retardation.

The MS displayed in Figure 2 was analyzed using an experimental measurement setup shown in **Figure** **3**a consisting of a 375 nm CW laser, two Glan‐laser calcite linear polarizers, and a pickoff window. To quantify birefringence, Jones Matrix analysis is used, where, for orthogonal linear polarizers indicated by LP_1_ and LP_2_, we have

(1) $$I_{1}=\;\sin^{2}\left(2\alpha \right)\;\sin^{2}\left(\frac{\Delta \Phi }{2}\right)I_{0}$$

with *I*_0_ being the incident beam intensity, *I*_1_ is the transmitted intensity, Δ*Φ* is the phase retardation between the fast and slow axis, and *α* is the angle with respect to the horizontal for the birefringent sample's fast axis. During measurements, the birefringent MS is rotated about the optical axis as indicated in the 3D view shown in Figure 3a. It is seen from Equation (1) that, for orthogonally positioned linear polarizers, rotation of the birefringent sample through angle *α* about the optical axis will result in a sinusoidal signal that can be fit to extract the phase retardation Δ*Φ*. Derivation of Equation (1) is contained in Supporting Information, and this measurement technique was used on commercially available quarter‐wave and half‐wave plates for validation.

Results from birefringence measurements for the MS, shown in Figure 2b, as it is rotated 90° about the optical axis are shown as the black data in Figure 3b. As expected, a sinusoidal signal was recorded; the fitting of this data according to Equation (1) yielded a measured phase retardation Δ*Φ* = 6.57 ± 0.05°. A reference fused silica slab (planar fused silica, i.e., no etched metasurface) was also measured, and the resultant signal is depicted in Figure 3b by the blue data. No measurable birefringence outside the noise level was observed. Using Equation (1), the expected signal for the cases of Δ*Φ* = 2.5° and 5° are shown as the red and magenta dashed lines, respectively, to demonstrate the trend with increasing phase retardation. The presence of a birefringent response by the etched structure demonstrates that glass‐engraved metasurfaces can be utilized for applications requiring polarization rotation. Furthermore, as structures fabricated through this etching mask approach have been previously demonstrated to exhibit a high laser damage threshold,^[^ ^29^ ^]^ this opens exciting possibilities of polarization rotation for high energy and high power laser applications. Combining polarized beam splitters and a waveplate provides a commonly used nonabsorbing attenuation element that is key for laser systems. Even more enticing applications may follow when the technology demonstrated here merges with the previously demonstrated ability to pattern the etching mask spatially,^[^ ^32^ ^],^ i.e., birefringent metaoptics with polarization rotation patterning.

**Figure 4:**
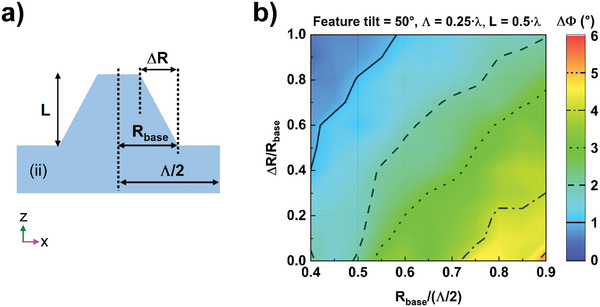
Modeled structure (in plane ii) and finite difference time domain (FDTD) simulation results for metasurfaces with angled features. Unit cell used for modeling the metasurface (MS) feature geometry‐induced birefringence (in plane ii) (a) and resultant FDTD simulation results (b). For the phase retardation contour plot (b), the features are tilted 50° with respect to the surface normal, the period (*Λ*) is a quarter of a wave, and the layer thickness *L* is half of the optical wavelength (*λ*).

To assist in mapping out the broad MS geometry parameter space, full‐wave simulations were performed; these simulations match the measured results. A finite difference time domain (FDTD) simulation of Maxwell Equations (Lumerical FDTD) modeling study was carried out to explain the performance dependence on the structure parameters; the MS feature geometry used in the simulations is presented in **Figure** **4**a while viewing plane (ii) as described in Figure 1c. The features are allowed to vary from cylinders (Δ*R*/*R*_base_ = 0) to cones (Δ*R*/*R*_base_ = 1), with a height *L*. The depiction in Figure 4a displays the simulation unit cell, with the expression *R*_base_/(*Λ*/2) representing a one‐dimensional view of the area coverage; the true area coverage, or fill factor, will be given by *R*_base_/(*Λ*/2)^2^. As Figure 4a is showing plane (ii), it is important to recall that in in the orthogonal direction of plane (i) these features are tilted with respect to the surface normal. This geometry is then fed in to a FDTD model, see Figure 4b, to predict the birefringence for a fixed feature tilt of 50° with respect to the surface normal, a period *Λ*  = 0.25*λ*, and layer thickness *L*  =  0.5*λ*, where *λ* is the wavelength at which operation is intended (here, 375 nm). It is seen, then, that for a given *R*_base_/(*Λ*/2) the birefringence increases as the features become more cylindrical, and for a given feature shape Δ*R*/*R*_base_ the birefringence increases with increasing feature area coverage. Or phrased differently, because *R*_base_ is determined by the etching mask, birefringence increases with increasing etching mask area coverage. For the MS with cylindrical features shown in Figure 2, *L*  =  450 nm and *R*_base_/(*Λ*/2) = 0.53; from these values the simulated phase retardation is 6.69°, which is in good agreement with the measured value of 6.57°.

**Figure 5:**
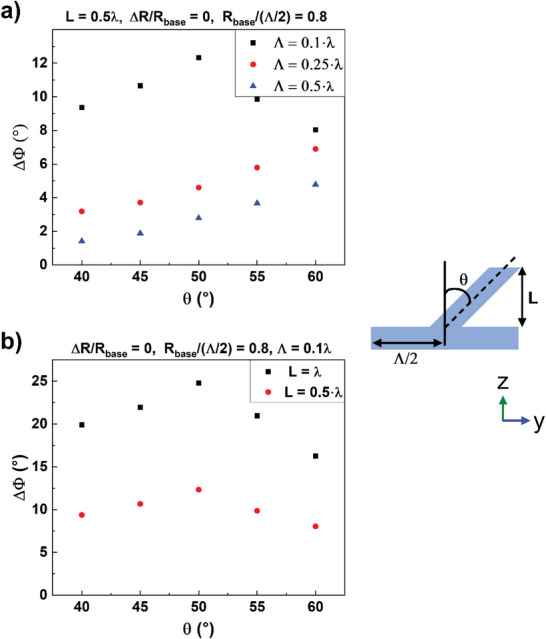
Roadmap to enhanced polarization retardation rotation: impact of etching mask period and metasurface (MS) layer thickness. Phase retardation angle (Δ*Φ*) as a function of the MS pillar tilt angle (*θ*): varying the MS period (a) and MS layer depth (*L*) (b).

While this demonstration is a first‐of‐its‐kind, further developing the structure to produce a higher birefringence is key to enabling many applications. Figure 4b gives a roadmap to increasing the birefringence by increasing the mask fill‐factor while maintaining a cylindrical feature geometry. As Figure 4 reveals that cylindrical features perform better at a tilting angle of 50°, FDTD calculations were carried out for cylindrical features with a constant MS feature height of 0.5*λ* and *R*_base_/(*Λ*/2) = 0.8, see **Figure** **5**a. The value *R*_base_/(*Λ*/2) =  0.8 was chosen for the practical reason that the corresponding masking fill factor, *R*_base_/(*Λ*/2)^[^ ^2^ ^]^ = 0.64, has already been demonstrated, refer to Supporting Information here. For the prescribed cylindrical features with layer thickness *L*  =  0.5*λ* shown in Figure 5a, as the period decreases from 0.5*λ* (blue triangles) to 0.1*λ* (black squares), the phase retardation increases from ≈2.5° to ≈12.5° at a tilting angle of 50° from the surface normal. While the cases of *Λ*  =  0.5*λ* and *Λ*  =  0.25*λ* continue to increase with increasing tilt angle, at this time we do not consider cases when *θ* > 60° due to the practical implication that, because of undesirable mask material removal during etching, steeper etching angles make it progressively more challenging to increase the layer thickness in the vertical direction.

To investigate the impact of increasing the MS layer thickness, cylindrical features with *R*_base_/(*Λ*/2) = 0.8 and *Λ*  =  0.1*λ* were used in simulations with *L* = 0.5*λ* (red circles) and *L* = *λ* (black squares) in Figure 5b. It is seen that, as the MS layer thickness increases by a factor of 2, for all tilt angles simulated the phase retardation increases by roughly a factor of two. This finding is intuitive, as each discrete layer should contribute ≈equally to the retardation.

The results from Figure 5 outline the roadmap from a birefringence of 6.6° (as reported here for a MS with period *Λ*  =  0.25*λ*, *L* = 1.2*λ*, and *R*_base_/(*Λ*/2) = 0.53) to a quarter‐wave by decreasing the period and increasing the MS layer thickness for a targeted 1/8 of a wave per optic surface. Notably, as seen in Figure 5b, a period of 0.1*λ* and layer thickness of 2*λ* will yield polarization rotation Δ*Φ*  = 50°. This indicates that an optic processed on both sides will surpass what is necessary to qualify as a quarter‐wave plate. If, however, it is technologically challenging to obtain a period of 0.1*λ*, Figure 5b reveals that MS layer thickness can be increased beyond 2*λ* as a means of compensation. This implies the capability of fabrication of a quarter‐wave plate that is monolithic to the underlying substrate and compatible with high power and energy lasers.

## 3 Structure for Manipulation of Water/Surface Interaction

**Figure 6:**
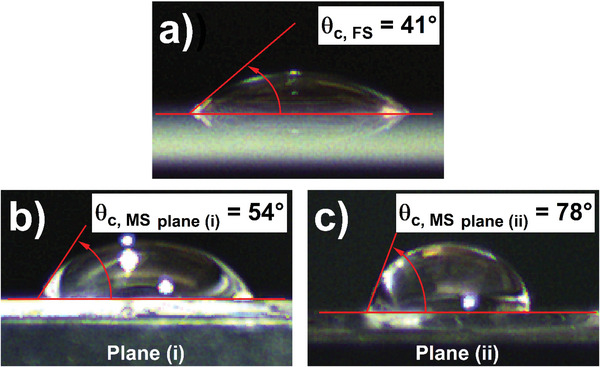
Metasurface (MS) topography influence on the water contact angle (*θ*_c_). Water contact angle on planar (unetched) fused silica (a) anisotropic MS plane (i) (b), and anisotropic MS plane (ii) (c). In all cases, 40 µL water was used.

Anisotropy in the MS structure lends itself to other applications, such as manipulation of the interaction between the etched structure and water. Static water contact angle (*θ*_c_) measurements were taken using a 40 µL droplet of water on reference (unetched) fused silica and an anisotropic MS with planes (i) and (ii) facing the camera; images of these are shown in **Figure** **6**a–c, respectively. For the reference fused silica, the contact angle was measured to be 41.2 ± 4.1°, and for the MS planes (i) and (ii) the static contact angles were 54.3 ± 6.1° and 77.6 ± 4.9°, respectively. This emphasizes a MS‐introduced anisotropy to the surface of an isotropic material. The MS contact angles, while still considered hydrophilic, represent a shift toward a hydrophobic structure (conventionally accepted to be *θ*_c_ > 90°). The fraction of substrate material in contact with the droplet can be determined from the Cassie–Baxter equation, $\cos\theta_{C}^{MS}=\varphi_{FS}\left(\cos\theta_{C}^{FS}+1\right)-1$, where $\theta_{C}^{MS}$ is the contact angle of water on the MS, $\theta_{C}^{FS}$ is the contact angle on the underlying fused silica, and $\varphi_{FS}$ is the contact area fraction between the water and fused silica MS, projected to the surface plane.^[^ ^38^ ^]^ From these measurements, the fractional contact area, $\varphi_{FS}$, for the two principal directions was found to be 90% along plane (i), and 69% along plane (ii). Manipulation of the MS period, area coverage, and/or feature tilt are expected to impact the wettability of these structures without any chemical modification to the MS beyond the etching process itself; however, this is a subject for future work.

**Figure 7:**
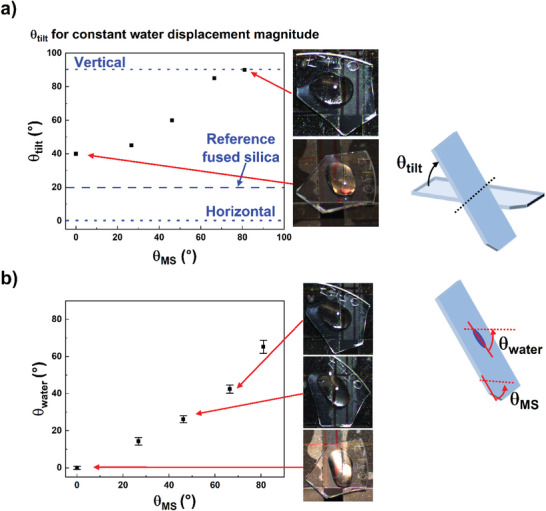
Water flow control and redirection by changing anisotropic metasurface (MS) feature orientation. Anisotropic MS was rotated in‐plane by angle *θ*_MS_ and then tilted in the vertical direction, *θ*_tilt_, until a 40 µL water droplet was displaced by a constant distance (a) and the angle, *θ*_water_, the water trajectory center line makes relative to the water center line of the *θ*_MS_  =  0° case as the MS is rotated in‐plane and then tiled (b).

Another usage for the angled structures fabricated here is to control and redirect the flow of water by modifying the orientation of the MS, i.e., orienting such that plane (i) from Figure 1 is parallel or orthogonal to the direction of gravity. As indicated in the diagrams on the right side of **Figure** **7**, the MS was placed horizontally and a 40 µL water droplet was placed on the surface. The sample was then tilted toward the vertical direction in increments of 5° at a rate ≈2.5° s^−1^, held at each incremental steps for 5 s, and the angle *θ*_tilt_ was recorded when the droplet had traveled a constant distance *l*. The MS was allowed to dry, and this procedure was repeated following rotation in the initial horizontal plane by angle *θ*_MS_, see Figure 7a. As the MS is rotated in‐plane by 81° relative to the initial orientation *θ*_MS_ = 0° (where *θ*_MS_ = 0° corresponds to viewing plane (ii) as shown in Figure 1), the tilting angle necessary for the water droplet to travel a constant length increases from *θ*_tilt_ = 40° at *θ*_MS_ = 0° to *θ*_tilt_ =  90° at *θ*_MS_ = 81°. This indicates that the effective propagation of water flow is prohibited in one direction and flows freely in the other principal direction, and is also consistent with the fractional coverages determined earlier for plane (i) and plane (ii). A piece of reference fused silica was measured in this way and exhibited a necessary tilting angle of 20°, which is 20° lower than the smallest angle recorded for the MS.

Given the tendency for water to flow preferentially along one of the principal axes, the directionality of the MS features can be harnessed to manipulate the path of water flow. As the MS is rotated from its original position to *θ*_MS_  =  81°, the direction of water flow is shown in Figure 7b as *θ*_water_, where *θ*_water_ is the angle the center line of water propagation makes with the water center line for the case of *θ*_MS_  =  0°. It is seen that the 81° rotation of the sample caused the direction of water flow to rotate 65 ± 4° relative to the initial direction. The findings presented in Figure 7a,b open the possibility of steering water to drain channels or inhibiting the flow of water altogether, among other potential applications. We should point out that all this work pertains to fused silica that has not been chemically modified beyond the initial etch itself; fabrication of a coating to create superhydrophobicity may amplify the characteristics shown here, further opening the door for self‐cleaning optics applications.

## 4 Conclusion

RIBE at an angle through metal etching masks has been demonstrated here as a fabrication process for generation of angled MS features. Anisotropic index of refraction for these MS enables nonbirefringent materials to exhibit birefringence. To demonstrate this, fused silica was etched at an angle of 48° with respect to the surface normal, producing a MS layer thickness of 450 nm. This structure was shown to exhibit polarization rotation of 6.57° under illumination by a 375 nm source. A simulation‐informed roadmap to obtaining a quarter‐wave plate via birefringent MS has been outlined. Additionally, the imparted anisotropy lends itself to manipulation of physical properties of the structure as well, with MS orientation suppressing water flow along one principal axis and giving rise to the capability of water flow steering. These findings pave a path to an engineering approach where the optical component material is selected for the desired optical/thermal/electrical properties, and through addition of a carefully engineered metasurface, anisotropy is obtainable. When taken in conjunction with the demonstrated capability of spatially patterning etching masks, the results presented here could enable metaoptics with patterned polarization rotation and engineered hydrophobicity/water steering, potentially altering the way optics are designed and conceptualized within optical systems.

## Conflict of Interest

The authors declare no conflict of interest.

## Supporting information

Supporting Information

## Data Availability

The data that support the findings of this study are available from the corresponding author upon reasonable request.
