# Supplementary material for: Birefringent Glass‐Engraved Tilted Pillar Metasurfaces for High Power Laser Applications
Source: Adv Sci (Weinh). 2023 Jun 19;10(24):2301111. doi: 10.1002/advs.202301111 (PMC10460841; doi:10.1002/advs.202301111)
Supplement: Supplementary file 1 — Supporting Information [file ADVS-10-2301111-s001.pdf]

ADVANCED SCIENCE
Open Access

Supporting Information

for *Adv. Sci.*, DOI 10.1002/advs.202301111

Birefringent Glass-Engraved Tilted Pillar Metasurfaces for High Power Laser Applications

Nathan J. Ray, Jae-Hyuck Yoo, Hoang T. Nguyen, Michael A. Johnson and Eyal Feigenbaum**

Birefringent Glass-Engraved Tilted Pillar Metasurfaces for High Power Laser Applications

NATHAN J. RAY*, JAE-HYUCK YOO, HOANG T. NGUYEN, MICHAEL A. JOHNSON, EYAL FEIGENBAUM**

Supplementary Information

Fused silica substrates purchased from Valley Design (2-inch diameter, 1 mm thick, 0.5 nm RMS) were used for this work. Prior to usage, all substrates were cleaned via a Piranha etching procedure.

Following cleaning, a Pt films were deposited via electron-beam deposition at a deposition rate of 1 $Å \cdot s^{-1}$. These films were then dewet by spatial invariant annealing in a furnace for 30 min at target setpoint; a montage of dewet structures as a function of film thickness and dewetting setpoint are shown in Figure S1. Analogous to work reported previously for gold on fused silica[1], at a constant dewetting temperature, increasing the initial metal film thickness results in an increase in the mean particle size and particle center-to-center spacing (period) and a decreased area coverage, or fill factor (FF). For a constant film thickness with increasing dewetting temperature, the period increases and the FF decreases, indicating more hemispherical-like particles that are taller in height.

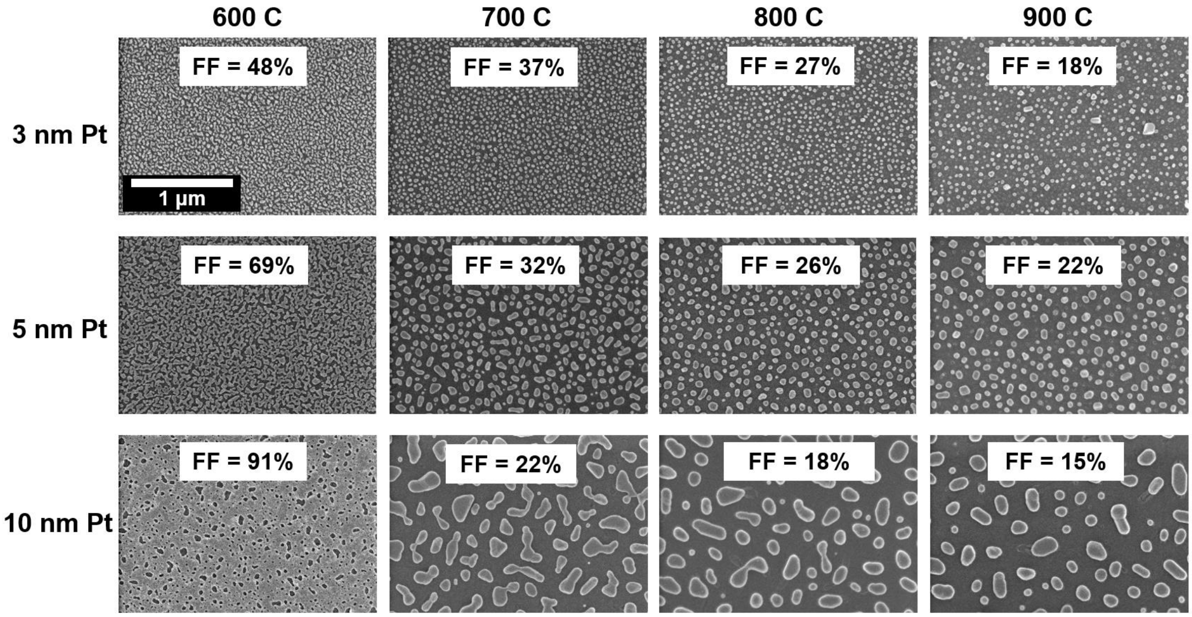

Figure S1. Montage of particles generated by dewetting Pt films. The rows correspond to different as-deposited films thicknesses, while columns correspond to different dewetting temperatures. The particle area coverage, or fill factor (FF), is given for each image, where the light color designates to metal and the darker color indicates underlying substrate. All images have the same scale bar shown in upper left image.

To fabricate taller nanoparticles to be used in the subsequent fabrication step as an etching mask, seeded dewetting was implemented. Seeded dewetting, reported previously for gold on fused silica[2], is a process of iteratively depositing and dewetting. The initial film thickness and dewetting temperature are chosen such that the required spatial length scales (nanoparticle size and period) are obtained. To that dewet nanoparticle ensemble,

another thin film (of thickness identical to the original thin film) is deposited, and subsequently dewet. During the iterative dewetting processes, the material accumulates at the original seeded locations. Three steps of this seeded dewetting process are depicted in Figure S2. The twofold advantages of seeded dewetting are that (1) the mean nanoparticle height increases, and (2) the FF increases. This enables optimization of the parameter space for increased phase retardation as described in Figure 4.

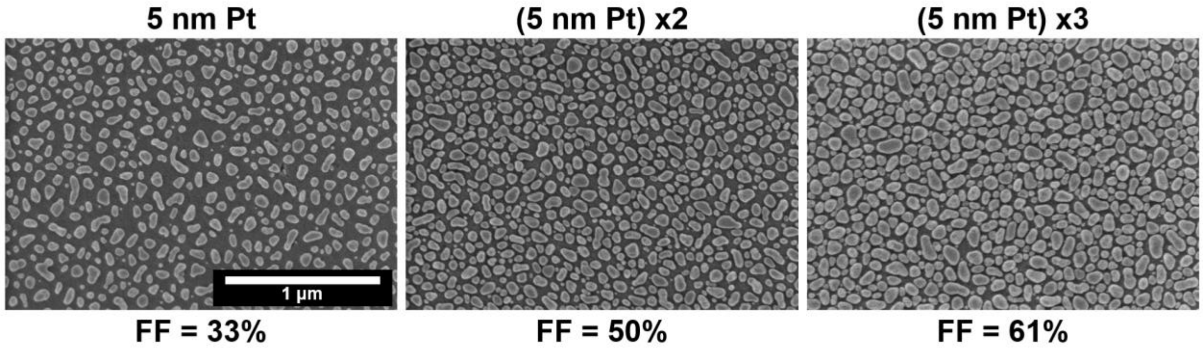

Figure S2. Nanoparticle ensemble fill factor increase as a function of seeded dewetting.

The underlying substrate was etched by reactive ion beam etching (RIBE) using CHF_3 to transfer the mask pattern to the substrate. To ensure no residual Pt following the etch, the optics were subjected to an aqua regia bath to remove any remaining platinum.

Reflectance measurements of structures similar to Fig. 2 (b) were measured and are depicted in Figure S3. Due to the anisotropic structure, there is a different index of refraction for the two principal directions. For a given polarization (here, P-polarization was used at an angle of incidence of 7°), the reflectance is sinusoidal with MS layer thickness as expected – MS 2 in Figure S3 is the best example of this. For cone-shaped features, the bandwidth of antireflective operation is increased. This has been well documented elsewhere.[3,4]

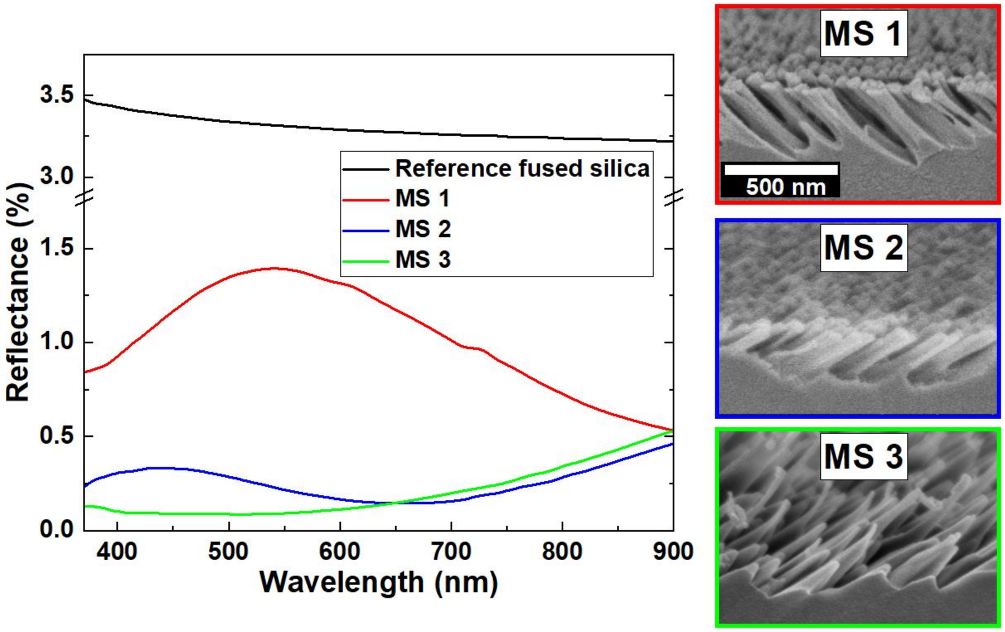

Figure S3. Reflectance as a function of wavelength for three etched structures with different MS feature geometries.

The extinction spectra of MS 2, given by the blue data from Figure S3, is shown in Figure S4. The curve is proportional to $1/\lambda^4$, indicating that scattering is dominant for shorter wavelengths. As these structures were fabricated from etching masks originating from 5 nm as-deposited Pt films, see Figure S1, the obvious solution to minimize scattering is to reduce the mask nanoparticle spacing, also demonstrated in Figure S1.

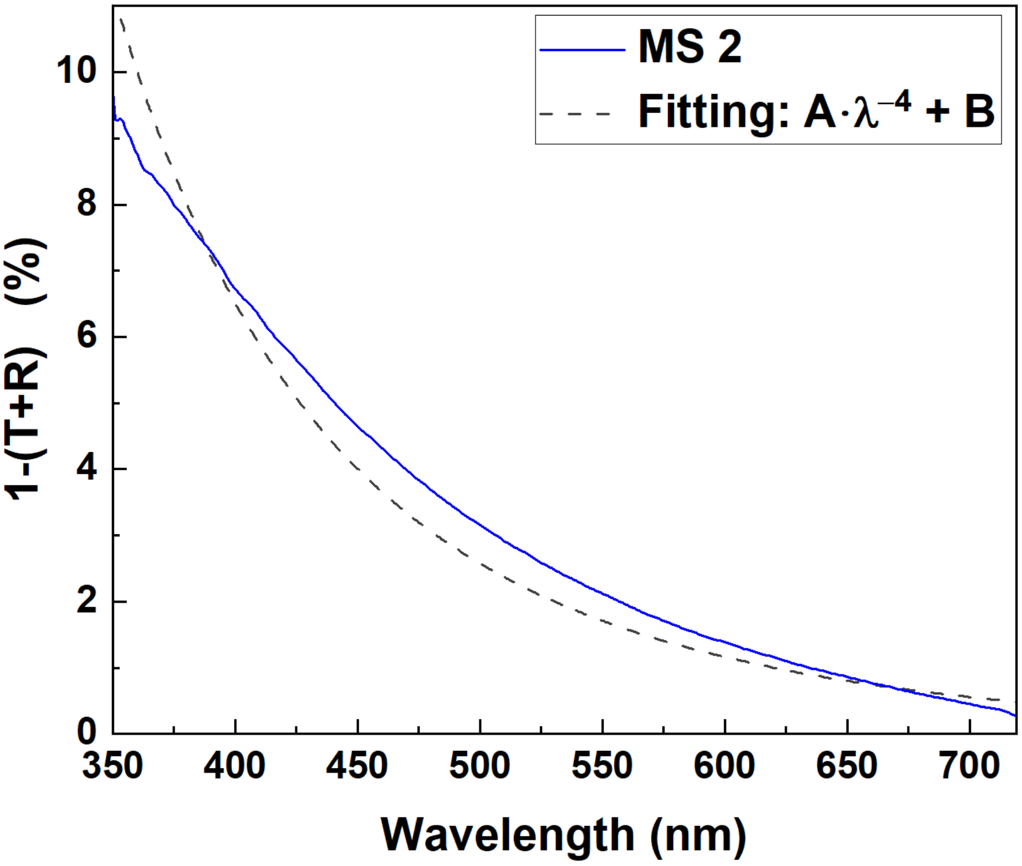

Figure S4. Extinction spectra of MS 2 from Fig. S3, given by the blue data.

The structure depicted in Fig. 2 (b) was analyzed using an experimental measurement setup shown in Fig. 3 (a) consisting of a 375 nm CW laser, two Glan-laser calcite linear polarizers, and a pickoff window. To quantify birefringence, Jones Matrix analysis is used. For orthogonally positioned polarizers indicated by LP_1 and LP_2 in Fig. 3 (a), we have

$$\overline{E_1} = \begin{pmatrix} 1 & 0 \\ 0 & 0 \end{pmatrix} \cdot R(-\alpha) \cdot \begin{pmatrix} 1 & 0 \\ 0 & e^{i\eta} \end{pmatrix} \cdot R(\alpha) \cdot \begin{pmatrix} 0 & 0 \\ 0 & 1 \end{pmatrix} \cdot \overline{E_0}\ , \tag{1}$$

where E_0 is the input polarization, E_1 is the output polarization, η is the phase retardation between the fast and slow axis, α is the angle with respect to the horizontal for the birefringent sample's fast axis, and R(δ) is the generalized rotation matrix

$$R(\delta) = \begin{pmatrix} \cos(\delta) & \sin(\delta) \\ -\sin(\delta) & \cos(\delta) \end{pmatrix} \tag{2}$$

Eq. 1 can be simplified to

$$\begin{bmatrix} E_{1,x} \\ E_{1,y} \end{bmatrix} = \begin{bmatrix} \{(1 - e^{i\,\eta}) \cos(\alpha) \sin(\alpha)\} E_{0,y} \\ 0 \end{bmatrix}, \tag{3}$$

where E_0 and E_1 have been broken down into orthogonal components. The norm of Eq. 3 will describe the intensity of transmitted light, I, as a function of α. Evaluation of this norm yields:

$$I_1 = sin^2(2\alpha)\, sin^2\left(\frac{\eta}{2}\right) I_0,$$

from which it follows that the ratio I_1/I_0 will be described by a sinusoidal signal at the metasurface is rotated angle α about its optical axis.

Optical measurements were carried out using the setup shown in Fig. 3 (a). To account for laser fluctuations, a pickoff was used for normalization purposes during all measurements. Reflectance loss introduced by the two polarizers and pickoff window was resolved by recording the signal-to-pickoff ratio when the polarization axes were aligned, i.e., the incoming power. The polarization axes were then positioned orthogonal to each other, the sample was inserted, and it was rotated about the optical axis to find the minimum throughput to detector #2 in Fig. 3 (a), i.e., matching the fast and slow axis directions to those of the polarizers. As the metasurface was rotated about the optical axis, power measurements from detectors #1 and #2 were recorded and their ratio was normalized against the previously recorded input power signal-to-pickoff ratio.

References

1 Ray, N. J. *et al.* Enhanced Tunability of Gold Nanoparticle Size, Spacing, and Shape for Large-Scale Plasmonic Arrays. *ACS Applied Nano Materials* **2**, 4395–4401 (2019).

2 Ray, N. J. *et al.* Tuning Gold Nanoparticle Size with Fixed Interparticle Spacing in Large-Scale Arrays: Implications for Plasmonics and Nanoparticle Etching Masks. *ACS Applied Nano Materials* **4**, 2733-2742 (2021). https://doi.org:10.1021/acsanm.0c03371

3 Feigenbaum, E., Ray, N. J. & Yoo, J.-H. Optical modeling of random anti-reflective meta-surfaces for laser systems applications. *Applied Optics* **58**, 7558 - 7565 (2019).

4 Ray, N. J., Yoo, J. H., Nguyen, H. T. & Feigenbaum, E. All-Glass Metasurfaces for Ultra-Broadband and Large Acceptance Angle Antireflectivity: from Ultraviolet to Mid-Infrared. *Advanced Optical Materials* (2023). https://doi.org:10.1002/adom.202300137
